# Work Experience, Job-Fulfillment and Burnout among VMMC Providers in Kenya, South Africa, Tanzania and Zimbabwe

**DOI:** 10.1371/journal.pone.0084215

**Published:** 2014-05-06

**Authors:** Linnea Perry, Dino Rech, Webster Mavhu, Sasha Frade, Michael D. Machaku, Mathews Onyango, Dickens S. Omondi. Aduda, Bennett Fimbo, Peter Cherutich, Delivette Castor, Emmanuel Njeuhmeli, Jane T. Bertrand

**Affiliations:** 1 Tulane University School of Public Health and Tropical Medicine, Department of Global Health Systems and Development, New Orleans, Louisiana, United States of America; 2 The Centre for HIV and AIDS Prevention Studies, Johannesburg, South Africa; 3 Zimbabwe AIDS Prevention Project, University of Zimbabwe College of Health Sciences, Department of Community Medicine UZ, Harare, Zimbabwe; 4 Jhpiego, Dar es Salaam, Republic of Tanzania; 5 FHI 360, Kisumu, Kenya; 6 Impact Research and Development Organization, Kisumu, Kenya; 7 Ministry of Health and Social Welfare, Dar es Salaam, Republic of Tanzania; 8 National AIDS/STI Control Programme, Nairobi, Kenya; 9 United States Agency for International Development, Washington, DC, United States of America; World Health Organization, Switzerland

## Abstract

**Background:**

Human resource capacity is vital to the scale-up of voluntary medical male circumcision (VMMC) services. VMMC providers are at risk of “burnout” from performing a single task repeatedly in a high volume work environment that produces long work hours and intense work effort.

**Methods and findings:**

The Systematic Monitoring of the Voluntary Medical Male Circumcision Scale-up (SYMMACS) surveyed VMMC providers in Kenya, South Africa, Tanzania, and Zimbabwe in 2011 (n = 357) and 2012 (n = 591). Providers self-reported on their training, work experience, levels of job-fulfillment and work fatigue/burnout. Data analysis included a descriptive analysis of VMMC provider characteristics, and both bivariate and multivariate analyses of factors associated with provider work fatigue/burnout. In 2012, Kenyan providers had worked in VMMC for a median of 31 months compared to South Africa (10 months), Tanzania (15 months), and Zimbabwe (11 months). More than three-quarters (78 – 99%) of providers in all countries in 2012 reported that VMMC is a personally fulfilling job. However, 67% of Kenyan providers reported starting to experience work fatigue/burnout compared to South Africa (33%), Zimbabwe (17%), and Tanzania (15%). Despite the high level of work fatigue/burnout in Kenya, none of the measured factors (i.e., gender, age, full-time versus part-time status, length of service, number of operations performed, or cadre) were significantly associated with work fatigue/burnout in 2011. In 2012, logistic regression found increases in age (p<.05) and number of months working in VMMC (p<.01) were associated with an increased likelihood of experiencing work fatigue/burnout, while higher career total VMMCs decreased the likelihood of experiencing burnout.

**Conclusion:**

Given cross-country differences, further elucidation of cultural and other contextual factors that may influence provider burnout is required. Continuing to emphasize the contribution that providers make in the fight against HIV/AIDS is important.

## Introduction

Scaling-up voluntary medical male circumcision (VMMC) to reach and maintain 80% coverage among men ages 15–49 in 13 priority countries would require performing 20.33 and 8.42 million circumcisions between 2011 – 2015 and 2016 – 2025, respectively [1]. This ambitious target requires significant human resource investments in settings where health systems and providers are already taxed [2]. The World Health Organization (WHO) emphasizes the importance of managing the entire “working lifespan,” or the professional cycle of health care providers from workforce entry, workforce performance, to workforce exit [3]. VMMC programs, in harmony with their respective health systems, must address workforce issues at every stage in the working lifespan by implementing strategies to (1) better train VMMC providers for workforce entry, (2) sustain and motivate providers to enhance performance and (3) retain providers by mitigating migration and attrition.

A major workforce challenge for VMMC programs is to identify and train enough qualified clinical personnel to perform the number of procedures necessary to reach the current targets and maximize the long-term population-level impact on HIV transmission. Three of the four countries participating in this study, Kenya, Tanzania, and Zimbabwe, have critical shortages of healthcare professionals (defined as not meeting a threshold of 2.5 health care professionals, physicians, nurses, and midwives, per 1,000 population) [3]. Tanzania has just.02 physicians per 1,000 population, followed by Kenya (.14) Zimbabwe (.16) and South Africa (.77). Similarly, Tanzania has just.37 nurses per 1,000 population followed by Zimbabwe (.72), Kenya (1.14) and South Africa (4.08) [3].

The development and effects of burnout on health care providers has been a health systems research interest for more than three decades [4]. In the 1990s, extensive research was conducted on the effect of HIV/AIDS work on health care providers as a marker of burnout; however this research was largely focused on the emotional impact of providing care to HIV/AIDS patients [5]. Research in the related area of the antiretroviral therapy scale-up has pointed to the importance of limiting burnout and attrition, in addition to maintaining providers’ level of job performance and satisfaction, to achieve maximum program efficiency [6]. Previous studies, in other settings, also show high monetary costs are associated with the decreased productivity and turnover caused by health care provider burnout [7]. The physical strain of standing for many hours, potential for monotony with repeating the same procedure in high volume overextended time periods, and emotional exhaustion, are thought to contribute to burnout and attrition among VMMC providers. Among highly trained physicians, there is an added fear that working in VMMC will result in the loss of their other clinical skills [8]. The potential impact of provider burnout on VMMC programs is high. One recent study conducted in Nyanza, Kenya, found that VMMC providers who had performed higher numbers of circumcisions achieved progressively lower rates of adverse events and shorter procedure times than their less experienced colleagues [9]. This substantiates the view that retaining experienced providers is an important factor for maintaining and improving the safety and efficiency of VMMC services. Knowing the characteristics of VMMC providers as well as their attitudes towards their work allows programs to improve workforce strategies.

This analysis examines this issue of performance and attrition in VMMC programs by looking descriptively at job-fulfillment and burnout. To date, little research has been done to profile VMMC providers or understand the effects of burnout on providers involved in the scale-up of VMMC. Specifically, in this analysis we will: 1) describe the profile and “working lifespan” of VMMC providers in the four SYMMACS countries (i.e., Kenya, South Africa, Tanzania and Zimbabwe) in terms of age, gender, training, role in the operating theater, and experience performing VMMC; 2) explore job-fulfillment, burnout, and the relationship between the two; and 3) identify factors associated with burnout among VMMC providers in Kenya.

## Methods

### Sampling

SYMMACS methodology has been described elsewhere in this supplement and in a detailed final report of the full study available online [10], [11]. In brief, this was a multi-country study of service delivery in four eastern and southern African countries scaling-up VMMC: Kenya, South Africa, Tanzania, and Zimbabwe. Two cross-sectional samples of VMMC sites were conducted in each country, the first between April and December 2011 (n = 73) and the second approximately 12 months later in 2012 (n = 122). In three countries (South Africa, Tanzania and Zimbabwe) where VMMC was being rolled-out for HIV prevention, the 2011 sample included all sites which were known to be providing VMMC services as part of the scale-up. However, by 2011, Kenya had a larger more developed VMMC program and sites were randomly sampled for inclusion into the study from a complete sampling frame of 235 VMMC sites. All sites visited in 2011 were revisited in 2012, with the exception of five sites in Kenya and eight in Zimbabwe which were no longer operational at the time of data collection in 2012. Replacement sites for Kenya were identified from the original sampling frame. In the other three countries, additional sites were selected in 2012 using purposeful sampling based on two objectives: 1) to sample high-volume sites in order to visit sites responsible for the largest proportion of VMMCs; 2) to maximize variation across sites in order to best reflect the individual patterns of VMMC scale-up in each country (by including sites across geographic region, different implementing partners or service delivery models) [10], [11]. The country teams developed their own specific selection criteria in order to meet these objectives. Across countries, all providers involved in the clinical aspects of VMMC service delivery on the two days of the site visit were invited to participate in the provider survey. In total n = 357 providers in 2011 (range 74–105) and n = 591 providers in 2012 (range: 82–209) took part in the study.

### Data collection

The social scientist on each team administered the provider questionnaire according to set data collection guidelines (used in all four countries). Interviews were generally conducted in English, except in Tanzania where the majority of providers were interviewed in Swahili. All interviews were conducted in visual and auditory privacy. In instances where the workload did not allow all eligible providers to be interviewed during the site visit, researchers made arrangements for the interviews to be conducted outside of working hours. The provider interview included a short structured survey and a series of open-ended questions on wide-ranging discussion issues including his/her attitude and perceptions about the VMMC scale-up.

### Variable Definition

Work fatigue and burnout was explored using the following questions from the survey tool: 1) “In your experience, have you noticed any provider fatigue/burnout among colleagues when they perform male circumcision full-time as a primary work activity?” This question served to introduce providers to the concept of burnout, and to help them use the experiences of their peers to prompt more genuine self-reflection when asked about their own experience of work fatigue/burnout; 2) “Performing (or assisting in performing) male circumcision is a personally fulfilling job;” and 3) “I personally have begun to experience work fatigue or burnout from performing (or assisting in performing) male circumcision repeatedly.” Provider responses were reported on a Likert scale representing the response types, “strongly agreed,” “agreed,” “neutral/didn’t know,” “disagreed,” or “strongly disagreed.” Work fatigue/burnout and job-fulfillment were dichotomized into “yes” if providers responded “strongly agree” or “agree” to the above questions/statements and “no” otherwise. The open-ended questions helped to contextualize the quantitative findings on this topic.

We defined “primary provider” as the clinician who is responsible for the critical steps of the procedure (removing the foreskin, achieving haemostasis, and applying the mattress sutures) [12]. National policy in South Africa and Zimbabwe dictates that a medical doctor must serve as primary provider, whereas in Kenya and Tanzania, other trained clinical providers (nurses, clinical officers, or assistant medical officers) can perform this role. The “secondary provider” – typically of a non-physician cadre – assists the primary provider with other steps in the VMMC procedure.

The duration of time which a provider has spent performing VMMC was calculated as the time difference from the interview and their initial VMMC procedure date (month and year). Providers were also asked to estimate the number of VMMCs they had performed during their professional career.

### Data Analysis

A descriptive analysis of all key provider characteristics included frequencies, means (presented with standard deviations) and medians (with interquartile ranges [IQR]) for continuous variables affected by outliers. Multiple regression analysis was not conducted to identify independent correlates of burnout across the four countries because of a strong “country effect” (variation across countries but not within countries on key variables). There was high collinearity between country and key explanatory variables in this analysis (e.g., provider cadre and role in surgical theater). Moreover, work fatigue or burnout was found to be most prevalent in one of the four countries: Kenya. Using bivariate analysis of data from Kenya, we examined whether provider gender, age, full-time versus part-time employment status, length of VMMC service provision (months), number of operations performed, role in surgical theater, or provider cadre were correlated with self-reported burnout. The bivariate analysis was conducted using the Pearson’s chi-square and Fisher’s Exact tests (expected cell frequencies were < 5). Multivariate logistic regression further explored these variables potential as predictors of work fatigue/burnout in Kenya separately for 2011 and 2012.

### Ethics approval

All study participants provided written consent and the consent forms were all approved by various IRB. USAID do not have Institutional Review Board but human subject approval was obtained through the Tulane University Institutional Review Board (IRB) and the local IRBs in each country; the Kenya Medical Research Institute, University of the Witwatersrand’s Human Research Ethics Committee in South Africa, Tanzanian National Institute for Medical Research, and the Medical Research Council of Zimbabwe. All those above-mentioned IRB approved the full study.

## Results

### Provider Profile

Table 1 shows the demographic characteristics of the VMMC providers, as well as their cadres and surgical roles. In Kenya (Nyanza Province, 70%) and Zimbabwe (67%) the majority of VMMC providers were male in 2012, whereas in South Africa (42%) and Tanzania (40%) males were the minority. However, in all countries where medical doctors provided VMMC, that cadre was predominately male. In 2012, in South Africa 25 of 31 physician providers were male, as well as 24 out of 25 in Zimbabwe and all 4 physicians in Tanzania were male. In 2012, the mean provider age was 31 in Kenya, in contrast to the other countries which ranged from 38 in Zimbabwe to 40 in Tanzania.

**Table 1:** VMMC provider profile in the four SYMMACS countries.

	Kenya		South Africa		Tanzania		Zimbabwe	
	2011	2012	2011	2012	2011	2012	2011	2012
	n = 85	n = 82	n = 105	n = 209	n = 93	n = 206	n = 74	n = 94
**Gender:**								
Male (%)	80.0	69.5	45.7	41.6	32.3	39.8	67.6	67.0
**Mean age in years,**	32.0	31.0	38.5	39.1	40.2	40.4	39.3	37.7
(Standard deviation)	(6.7)	(6.6)	(9.8)	(10.9)	(9.4)	(8.8)	(8.4)	(8.7)
**Cadre:**								
Medical Doctor	0.0	0.0	20.0	16.7	0.0	1.9	25.7	26.6
Nurse	52.9	53.7	80.0	83.3	80.6	78.6	74.3	73.4
Assistant medical officer (AMO)	0.0	0.0	0.0	0.0	8.6	5.3	0.0	0.0
Clinical officer	47.1	45.1	0.0	0.0	10.8	14.1	0.0	0.0
**Role in surgical theater, % providers that:**								
Primary provider ^A^	0.0	1.2	15.2	11.0	47.3	.5	25.7	26.6
Secondary provider ^B^	1.2	7.3	71.4	78.5	11.8	.5	74.3	73.4
Both perform and assist with VMMC operations depending on need	98.8	91.5	13.3	10.5	40.9	99.0	0.0	0.0
**Estimated number of VMMC procedures performed or assisted (career total):**								
Median	2430	1343	600	500	700	1500	360	400
(Interquartile range)	(500–4745)	(200–4185)	(200–2000)	(100–1000)	(275–1950)	(600–3000)	(70–1125)	(158–1000)
**Number of months performing VMMC for HIV prevention**								
Median	25 mo.	31 mo.	8 mo.	10 mo.	12 mo.	15 mo.	6 mo.	11 mo.
(Interquartile range)	(12–40)	(16–43)	(4–14)	(4–16)	(8–16)	(10–26)	(1–20)	(5–17)
**In the past 3 months % providers that performed VMMC:**								
Full-time ^C^	64.7	45.1	80.0	78.5	1.1	50.0	33.8	13.8
**% of providers that received:**								
VMMC training in medical or nursing school	36.5	20.7	20.0	4.3	7.5	1.0	4.1	4.3
Additional formal training/continuing education (e.g., certificate training) in VMMC for HIV prevention	98.8	97.6	76.7	75.1	97.8	100	100	100
**Among those who had additional training:**	**n = 85**	**n = 82**	**n = 105**	**n = 160**	**n = 91**	**n = 206**	**n = 74**	**n = 94**
Mean number of days of additional training	21.2 days	20.8 days	5.7 days	5.7 days	13.9 days	11.5 days	6.8 days	7.0 days
(Standard deviation)	(20.5)	(13.9)	(3.5)	(2.8)	(5.2)	(1.6)	(1.5)	(0.2)

[A] Primary providers perform VMMC (removes foreskin).

[B] Secondary providers assist the primary surgical provider.

[C] full time defined as dedicated ≥90% of working hours to VMMC.

### Provider training, role in surgical theater, and experience performing VMMC

Workforce structure in each country was reflective of national task-shifting and task-sharing policies. In 2011, the majority of providers in Tanzania had specific surgical roles (47% only performed VMMC and 12% only assisted, Table 1). However, in 2012, 99% of providers were both performing and assisting with VMMC depending on need. Although task-shifting is not authorized in South Africa, the SYMMACS data showed some evidence that it may occur, especially in high demand periods: 13% of nurses in 2011 and 11% in 2012 reported to have performed VMMC depending on need. However, in Zimbabwe all nurses served only as secondary providers and all medical doctors served exclusively as primary providers.

The percentage of providers working full-time (at least 90% of working hours) in VMMC ranged across countries, from Zimbabwe (14%), Kenya (45%), Tanzania (50%), and South Africa (79%). While on the opposite ends of the spectrum, in both South Africa and Zimbabwe physician providers were less likely to work full-time than their nurse colleagues: in South Africa (2012) 40% of MDs worked full-time in VMMC compared to 86% of nurses; in Zimbabwe (2012), 0% of MDs and 19% of nurses worked full-time. In Tanzania we saw a shift towards full-time work in VMMC, from just 1.1% of providers in 2011 to 50% of providers in 2012. The opposite pattern occurred in Zimbabwe where the percent of providers working full time in VMMC dropped from 33.8% to 13.8%.

Table 1 shows the training profiles of providers in each of the four countries. In Kenya, which has the longest running VMMC program, 21% of providers received training in VMMC from medical or nursing school as of 2012. However, training in medical and nursing school for VMMC was virtually non-existent in all other countries (1–4%). By 2012, three of the four countries were able to reach 98–100% of providers with additional formal training in VMMC for HIV prevention. The exception was South Africa, which had reached just 75% in 2012 (see Rech et al. in this supplement [13]). Among providers who received additional training, the mean number of days of additional training varies from about 21 days in Kenya (where providers had often participated in several trainings) to about 6 days in South Africa.

In Kenya (2012) the median time a provider had worked in VMMC was 31 months (IQR 16–43 months), which was more than twice the median time in any of the other countries. The median time worked was 10 months (IQR 4–16) in South Africa, 11 months (IQR 5–17) in Zimbabwe, and 15 months (IQR 10–26) in Tanzania. The median estimated number of procedures ranged from 1,500 (Tanzania), followed by 1,343 (Kenya), 500 (South Africa), and 400 (Zimbabwe). In Kenya (2012), 29% of providers also estimated that they had performed or assisted in over 3,000 procedures to date, as compared to 21% in Tanzania, 9% in Zimbabwe, and 6% in South Africa.

### Levels of provider job-fulfillment and work fatigue/burnout

Distributions of the responses to the work fatigue/burnout and job-fulfillment questions are shown in Table 2. The percent reporting to have observed provider fatigue/burnout among colleagues varied markedly by country. In 2012, 89% of Kenyan providers reported provider fatigue/burnout among colleagues occurs “frequently or occasionally,” followed by 49% of South African responders, 36% of Zimbabweans, and no Tanzanians. Providers who reported to have noticed any level of burnout among colleagues were asked how long on average it took for providers to begin to experience burnout as a result of working in VMMC. Over half of respondents across countries in both 2011 and 2012 reported that the time it took for work fatigue/burnout to appear depended on client volume and seasonal changes in workload. Specific references were made to increased burnout during winter/school holidays in South Africa and Zimbabwe, as well as high volume campaigns in Tanzania and Kenya (where they are called the Rapid Results Initiative or RRI) (data not shown).

**Table 2:** Job-fulfillment and work fatigue/burnout among VMMC providers by country.

	Kenya		South Africa		Tanzania		Zimbabwe	
	2011	2012	2011	2012	2011	2012	2011	2012
	n = 85	n = 82	n = 105	n = 209	n = 93	n = 206	n = 74	n = 94
**% providers who report noticing provider fatigue/burnout among colleagues when they perform VMMC full-time**								
Frequently	8.2	11.0	14.3	8.6	0.0	0.0	9.5	7.4
Occasionally	80.0	78.0	26.7	40.7	8.6	0.0	24.3	28.7
Very rarely	7.1	6.1	15.2	6.7	16.1	16.5	32.4	17.0
Not at all	4.7	4.9	41.0	41.6	74.2	82.5	29.7	46.8
Don’t know	0.0	0.0	2.9	2.4	1.1	1.0	4.1	0.0
**% Self-reported job-fulfillment**	87.1	84.0	82.9	79.9	100.0	99.0	81.1	77.7
**% Self-reported work fatigue or burnout**	70.6	65.9	36.2	32.5	53.8	14.6	27.0	17.0

When asked to report on their own job-fulfillment, providers were positive; the percentage of providers that agreed that performing VMMC is a personally fulfilling job ranged from 78% in Zimbabwe to 99% in Tanzania in 2012. However, there were large differences in the levels of self-report work fatigue/burnout across countries. In Kenya, 66% of providers had personally begun to experience work fatigue or burnout. Much lower levels were reported among providers in South Africa (33%), Zimbabwe (17%), and Tanzania (15%).

### Relationship between job-fulfillment and work fatigue/burnout

Providers who reported VMMC to be a personally fulfilling job generally had comparable levels of burnout, see Table 3. In Tanzania, work fatigue/burnout was still present despite 99–100% of providers reporting personal job-fulfillment. In South Africa (2012), there was a statistically significant association between lower levels of self-reported work fatigue/burnout and job-fulfillment: 26.9% of providers reporting job-fulfillment also reported work fatigue/burnout, as compared to 54.8% among those who did not report job-fulfillment (p<.001). In Kenya, job-fulfillment seemed to have a slight protective effect in 2011 but showed no association the following year. Similarly, no significant association was found either year in Zimbabwe.

**Table 3:** Association of VMMC provider job-fulfillment and work fatigue/burnout**.**

	Kenya				South Africa				Tanzania				Zimbabwe			
	2011		2012		2011		2012		2011		2012		2011		2012	
	% burnout		% burnout		% burnout		% burnout		% burnout		% burnout		% burnout		% burnout	
	n =	%	n =	%	n =	%	n =	%	n =	%	n =	%	n =	%	n =	%
**Provider reports job-fulfillment**																
Yes	74	66.2*	68	66.2	87	32.2	167	26.9***	93	53.8	203	13.8	60	28.6	73	13.7
No/neutral/don’t know	11	100*	13	61.5	18	55.6	42	54.8***	0	--	2	50.0	14	26.7	21	28.6
**Total**	85	70.6	82	65.9	105	36.2	209	32.5	93	53.8	205	53.8	74	27.0	94	17.0

* P<.05, **p<.01, ***p<.001, using the Pearson’s chi-square test statistic.

Providers consistently emphasized that fulfillment stemmed primarily from the knowledge that they were working for HIV prevention. This was also consistent with the qualitative data from the open-ended questions. When providers were asked how they felt about the VMMC scale-up in their countries, they frequently expressed pride for the work they were doing in their communities. However, when asked about the impact of the scale-up on their own work, the themes of unrealistic targets, increased workload, and fatigue were common.

### Factors associated with work fatigue/burnout in Kenya

No variable tested in the bivariate analysis was significantly associated with work fatigue/burnout in Kenya in 2011. However, the 2012 data showed that work fatigue/burnout significantly increased with provider age (p <0.05) and those who both preformed or assisted in VMMC depending on need were more likely to experience burnout (p<0.01) than providers who only assisted in VMMC (Table 4). Equivalent levels of burnout were found among males and females, clinical officers and nurses, and full and part-time VMMC providers, as well as across categories for length of employment in VMMC and number of VMMCs performed.

**Table 4:** Bivariate analysis of correlates of work fatigue/burnout in Kenya in 2011 and 2012.

	2011		2012	
I have personally begun to experience burnout:	n = 85	% yes	n = 82	% yes
**Gender of Provider**				
Male	68	73.5	57	68.4
Female	17	58.8	25	60.0
**Age of provider**				
18–34	62	64.5	62	68.1*
35–44	13	92.3	16	87.5*
45+	6	66.7	4	100*
**Cadre of provider**				
Clinical officer	40	77.5	37	64.9
Nurse	45	64.4	44	68.2
**Role in surgical theater**				
Perform circumcision as the primary provider (removes foreskin)	0	--	1	0.0**
Assist the surgical provider (secondary provider)	1	100	6	16.7**
Both perform and assist with VMMC depending on need	84	70.2	75	70.7**
**VMMC employment status**				
Full-time (≥ 90% of working hours in VMMC)	55	76.4	37	62.2
Part-time	30	60.0	45	68.9
**Number of VMMC procedures performed or assisted (career total)**				
1–100 VMMCs	8	62.5	14	78.6
101–500 VMMCs	14	64.3	17	64.7
501–1000 VMMCs	7	71.4	7	85.7
1001–3000 VMMCs	18	66.7	20	65.0
3001+	38	76.3	24	54.2
**Number of months of experience performing VMMC for HIV prevention**				
0–6 months	11	81.8	13	53.8
7–12 months	11	45.5	3	33.3
13–24 months	16	56.3	21	61.9
25+ months	47	78.7	45	73.3
**TOTAL**	**85**	**70.6**	**82**	**65.9**

* p <0.05, ** p <0.01, ***p <0.001, using Pearson’s chi square, except in cases of small cell frequencies (< 5 cases per cell) which use the Fisher Exact.

Multivariate logistic regression on work fatigue/burnout in Kenya (Table 5) tested the relative strength of the predictors explored in the bivariate analysis. Separate analyses were conducted for both 2011 and 2012. Role in surgical theater did not hold its significance in the regression and was dropped (along with provider cadre) from the final models. None of the explanatory variables yielded significant findings for 2011. In 2012, provider age (continuous) was a significant predictor of work fatigue/burnout (p = .027). Marginal effects show that on average each additional year of age is associated with a 2.7% increased likelihood of reporting work fatigue/burnout. Full-time versus part-time VMMC employment and gender were not significant predictors in either 2011 or 2012. The number of months worked in VMMC for HIV prevention was a significant predictor of work fatigue/burnout in 2012 (p = .004), with each additional month increasing the likelihood of experiencing work fatigue/burnout by an average of 1.2%. However, providers who had performed a higher number of career total VMMCs (1001–3000 VMMCs or 3001+ career VMMCs) had a significantly decreased likelihood of experiencing work fatigue/burnout over the reference category (1–100 VMMCs), p = .011 and p = .001 respectively. Controlling for months worked and the other factors, on average providers in the highest category (3001+ VMMCs) were 56.5% less likely to report work fatigue/burnout.

**Table 5:** Multivariate logistic regression: work fatigue/burnout in Kenya in 2011 and 2012.

	2011				2012			
	Coef.	LowerCI (95%)	Upper CI (95%)	P-value	Coef.	Lower CI (95%)	Upper CI (95%)	P-value
**Gender**								
Male	(ref)	--	--	--	(ref)	--	--	--
Female	–0.41	–1.67	0.84	0.519	0.03	–1.23	1.30	0.958
**Age (years)**	0.08	–0.01	0.18	0.094	0.17	0.02	0.33	0.027
**VMMC employment status**								
Part-time	(ref)	--	--	--	(ref)	--	--	--
Full-time (≥ 90% of working hours in VMMC)	1.05	–0.29	2.38	0.123	0.45	–0.82	1.72	0.486
**Number of VMMC procedures performed or assisted (career total)**								
1–100 VMMCs	(ref)	--	--	--	(ref)	--	--	--
101–500 VMMCs	0.88	–1.22	2.99	0.412	–1.81	–3.71	0.10	0.063
501–1000 VMMCs	0.73	–1.75	3.22	0.563	–2.36	–5.46	0.75	0.137
1001–3000 VMMCs	0.49	–1.59	2.57	0.643	–3.12	–5.53	–0.72	0.011
3001+	0.69	–1.47	2.86	0.531	–4.85	–7.63	–2.08	0.001
**Number of months of experience performing VMMC for HIV prevention**	0.00	–0.03	0.02	0.734	0.08	0.02	0.13	0.004
Constant	–2.83	–6.73	1.07	0.155	–4.06	–8.42	0.30	0.068

## Discussion

Human resource constraints in Sub-Saharan Africa have been identified as one of the major factors limiting the scale-up of VMMC [14]. This study helps to characterize VMMC providers and their professional profiles. To the authors’ knowledge, this is also the first exploration of job-fulfillment and work fatigue/burnout among health care providers involved in the VMMC scale-up.

The national profiles of VMMC providers are largely influenced by health workforce policy and programs will benefit from seeing the spectrum of staffing strategies. In Kenya and Tanzania, nearly all providers are non-medical doctors who receive specialized formal training in VMMC, while programs in South Africa and Zimbabwe rely entirely on a low-supply of medical doctors for primary providers. In Tanzania a shift toward full-time work in VMMC is the result of the official national scale-up, which has allowed sites to allocate more dedicated VMMC staff within larger health facilities. The opposite pattern occurred in Zimbabwe, where national scale-up has resulted in an increased number of public sector sites and outreach teams composed of public sector providers (both nurses and doctors) who are engaged in VMMC on a locum basis. This trend is similar in South Africa, except nurses tend to form part of a dedicated VMMC staff while physicians often operate on a part-time or locum basis. Program efforts to recruit, sustain and retain the health workforce must consider these differences in staffing strategies. Task-shifting policies emerge as an important strategy to overcome human resource constraints to VMMC scale-up.

Whereas one might hypothesize that job-fulfillment and burnout are opposite ends of a single dimension, the findings from this research show they are in fact independent constructs. Although VMMC providers in all four countries reported high levels of job-fulfillment, they were not immune to work fatigue or burnout. In Kenya, the country where work fatigue/burnout was most prevalent, bivariate and multivariate analysis provided some insight into possible predictors of work fatigue/burnout. However, in 2012, increases in the number of months worked in VMMC increased the likelihood of work fatigue/burnout among providers, while having preformed higher numbers of VMMCs was associated with a decreased likelihood of burnout. Provider turnover and other unmeasured contextual factors are likely confounding this relationship. The widespread nature of work fatigue and burnout identifies a need for a more in-depth treatment of this subject in future research, including qualitative methods.

### Limitations

SYMMACS was designed to track the natural evolution of VMMC scale-up. Therefore, the study planned to accommodate the addition of new sites and the sampling in each country was adjusted accordingly. However, this also emerges as a limitation of this study being as the result was a non-random sample of providers and variations in the sampling strategies across countries yielded imbalanced sample sizes.

The questions in this study pertaining to burnout were an attempt to explore self-reported level of work fatigue experienced by VMMC providers. The terms “work fatigue” and “burnout” were self-defined by providers and results are limited by the providers’ personal understanding of the terms. Moreover, in Tanzania, there appears to have been some problem with the translation of the word to Swahili, especially in relation to observing burnout among colleagues (0% of providers reported frequent or occasional burnout among colleagues in 2012). The concept of work fatigue/burnout could be interpreted differently across cultures or settings. In future studies, inclusion of an operational definition of burnout in the questionnaire or delivery of an established quantitative burnout inventory, such as the Maslach Burnout Inventory, could give a more objective picture of VMMC provider burnout [15]. Additionally, SYMMACS only included current VMMC providers and did not yeild data on health worker retention and workforce turnover which may have confounded the multivariate results.

## Conclusions

Mathematical modeling has shown that the rate at which VMMC coverage is achieved will greatly impact both the number of HIV infections averted and the cost-effectiveness of the intervention, therefore the speed of the roll-out and efficiency of services are vitally important [1]. The ability of governments to meet the ambitious VMMC targets with quality services will depend on retaining experienced providers that are willing to complete large numbers of VMMC procedures in high volume settings.

This exploratory study into the work fatigue and burnout experienced by VMMC providers documents a phenomenon that program managers will need to consider as the scale-up intensifies. Continuing to emphasize the contribution that providers make in the fight against HIV/AIDS is important. Given cross-country differences, further elucidation of cultural and other contextual factors that may influence work fatigue or burnout is required. Previous research suggests that multifaceted interventions which consider a variety of factors, such as motivation and job satisfaction, training, and supportive supervision may be most successful in improving health worker performance [16]. It will be useful for each scale-up country to consider how best to continue to support and motivate its VMMC providers in order to maintain job-fulfillment, reduce burnout, prevent attrition, and maximize performance.
